# Oral Pathogenic Bacteria-Inducing Neurodegenerative Microgliosis in Human Neural Cell Platform

**DOI:** 10.3390/ijms22136925

**Published:** 2021-06-28

**Authors:** Van Thi Ai Tran, You Jung Kang, Hyun-Kyoung Kim, Hyung-Ryong Kim, Hansang Cho

**Affiliations:** 1 Department of Biophysics, Institute of Quantum Biophysics, Sungkyunkwan University, Suwon 16419, Korea; ttaivan@g.skku.edu; 2 Department of Intelligent Precision Healthcare Convergence, Institute of Quantum Biophysics, Sungkyunkwan University, Suwon 16419, Korea; 3 Department of Mechanical Engineering and Engineering Science, University of North Carolina at Charlotte, Charlotte, NC 28223, USA; ykang15@uncc.edu; 4 School of Pharmacy, Jeonbuk National University, Jeonju 54896, Korea; khkyoung11@jbnu.ac.kr; 5 College of Dentistry, Dankook University, Choenan 31116, Korea

**Keywords:** Alzheimer disease, microgliosis, neurodegeneration, *Porphyromonas gingivalis*, pathogenic bacteria

## Abstract

*Porphyromonas gingivalis* is a gram-negative bacterium found in the human oral cavity and is responsible for the development of chronic periodontitis as well as neurological diseases, including Alzheimer’s disease (AD). Given the significance of the roles of *P. gingivalis* in AD pathogenesis, it is critical to understand the underlying mechanisms of *P. gingivalis*-driven neuroinflammation and their contribution to neurodegeneration. Herein, we hypothesize that *P. gingivalis* produces secondary metabolites that may cause neurodegeneration through direct or indirect pathways mediated by microglia. To test our hypothesis, we treated human neural cells with bacterial conditioned media on our brain platforms and assessed microgliosis, astrogliosis and neurodegeneration. We found that bacteria-mediated microgliosis induced the production of nitric oxide, which causes neurodegeneration assessed with high pTau level. Our study demonstrated the elevation of detrimental protein mediators, CD86 and iNOS and the production of several pro-inflammatory markers from stimulated microglia. Through inhibition of LPS and succinate dehydrogenase in a bacterial conditioned medium, we showed a decrease in neurodegenerative microgliosis. In addition, we demonstrated the bidirectional effect of microgliosis and astrogliosis on each other exacerbating neurodegeneration. Overall, our study suggests that the mouth-brain axis may contribute to the pathogenesis of AD.

## 1. Introduction

Alzheimer’s disease (AD) is the most common progressive neurodegenerative disease with clinical symptoms, such as memory loss at an early stage, that lead to a decline in the ability to respond to the living environment. Approximately 130 million people worldwide are estimated to be at risk of AD in 2050 according to the World Alzheimer Report 2015. Since the discovery of AD more than 100 years ago, several pathogenic mechanisms of AD have been proposed and the most recognized hypotheses relate to two distinctive protein markers, amyloid-beta (Aβ) and tau [1]. Moreover, oxidative stress and neuroinflammation have been proposed as the central mechanisms of neurodegeneration in AD [2].

A number of studies have demonstrated the connection between AD and neuroinflammation [3]. Inflammation and degeneration of brain cells that occur in the central nervous system have been linked to the accumulation of prion protein, including Aβ and pTau, which are two known markers of AD [4]. Microglia and astrocytes are the most abundant brain immune cells and mainly contribute to neuroinflammatory processes in neurodegenerative diseases [5]. Neuroinflammation in AD is associated with M1/M2 stage alterations in activated microglia, which are the major immune cells in the central nervous system [6]. Activated microglia cells change their morphologies, producing several proteins, pro-inflammatory cytokines, nitric oxide and reactive oxygen species [7]. 

Bacteria-mediated neuroinflammation has been considered a critical factor in neurodegeneration [8]. Bacterial lipopolysaccharide (LPS) and Aβ are two well-known factors that can trigger chronic neuroinflammation, resulting in neurodegeneration related to neuronal death and astrogliosis [9]. Several bacterial species and their metabolites are mediators of neurodegenerative diseases [10] and, hence, the determination of specific bacterial species or bacteria-derived factors is essential to explore the role of bacteria in AD. 

*Porphyromonas gingivalis*, commonly called gum bacteria, is an oral gram-negative anaerobe and a major pathogen of chronic periodontitis that is popular in both developed and developing countries, infecting approximately 20–50% of the global population with a high prevalence in adults and older people [11]. In a cohort study, chronic periodontitis patients had higher risks of dementia and AD than non-infected persons, which demonstrated the correlation of AD and periodontitis [12]. In addition, it has been reported that oral *P. gingivalis* treatment induced Aβ1-42 levels, which are oligomeric forms of amyloid plaques, a well-known cause of AD and that small molecules called gingipains, which are toxic proteases, are associated with AD pathology [13]. Immunofluorescence labeling of LPS from *P. gingivalis* was conducted for AD brain specimens, which have contributed to understanding the relationship between gum bacteria and AD [14]. Current approaches have only been able to determine the effect of *P. gingivalis* on neuronal cells, but it is not clear if bacteria damage neurons directly or indirectly through activated microglia. 

Therefore, we hypothesized that metabolites from *P. gingivalis* may trigger microgliosis and astrogliosis, which induce neuroinflammation, leading to neurodegeneration. In this study, we investigated the inflammatory roles of bacterial conditioned media in the activation of microglia and reactive astrocytes, which causes microgliosis and astrogliosis, as defined by immunostaining against several protein markers and measuring cytokines/chemokines released from stimulated cells. Our study demonstrated neurodegeneration by direct treatment with bacteria or indirectly through microgliosis to illustrate which mediators, bacteria or activated microglia, play a priority role in neurodegeneration. We also indicated the bidirectional interaction of microgliosis and astrogliosis and assessed microglial migration in brain-on-chip. Finally, we screened new metabolites from bacterial-conditioned media (BCM) as potential factors involved in neuroinflammation and demonstrated their cellular mechanisms. 

## 2. Results

### 2.1. Neurodegeneration Derived from Microgliosis and Astrogliosis-Induced Bacteria

**Figure 1:**
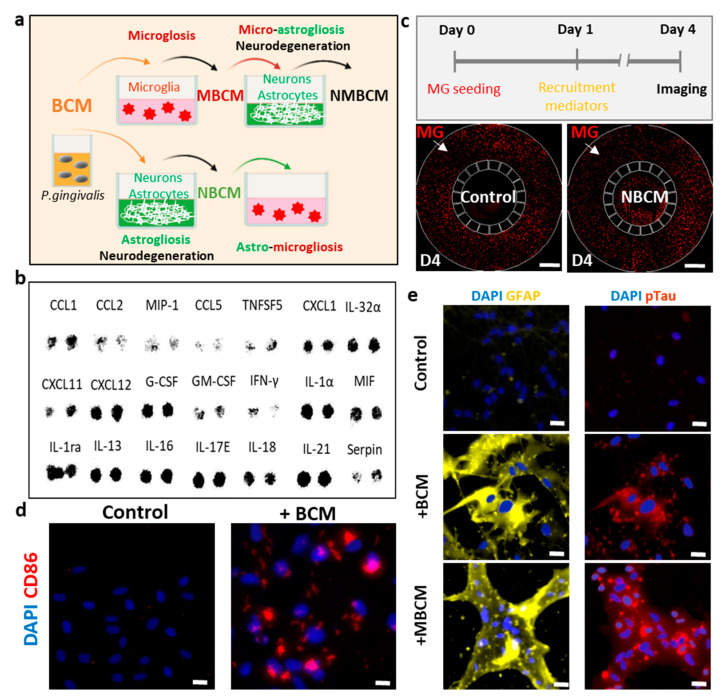
Utilization of human neural cells platforms to study neurodegenerative microgliosis induced by *P. gingivalis* infection. (**a**) Schematic of experimental design for assessment of microgliosis, astrogliosis and neurodegeneration under long-term exposure to bacterial conditioned media (BCM). (**b**) Multiple cytokines were detected in high-concentration BCM (BCM_H_). (**c**) Representative results showing microglial recruitment on chip (Scale bar, 500 μm). (**d**) Representative images showing pro-inflammatory microglial marker (CD86) in control and BCM-stimulated microglia, scale bar 50 μm. (**e**) Representative images for reactive astrocytes (GFAP), neurodegenerative marker (pTau) of neurons/astrocytes in control, BCM and MBCM stimulation, scale bar 50 μm.

To investigate the effects of bacterial metabolites on innate immunity in the brain, we treated microglia, astrocytes and neurons isolated from patients with chronic periodontitis with *P. gingivalis* and assessed microgliosis, astrogliosis and neurodegeneration (Figure 1a). It should be noted that we employed two independent platforms, single-culture microglia and co-cultured neurons and astrocytes, to investigate neuroinflammation and neurodegeneration induced by BCM-stimulated microgliosis and astrogliosis.

For the first platform and to investigate the effect of BCM on the microglia directly, we seeded human adult microglia (SV40) in a single-culture platform and treated them with a mixture of microglial media and BCM at a ratio of 10:1 (10 MM:1 BCM_L/H_) for 3 days and investigated the microglial cell response. We immunostained microglia with antibodies targeting triggering receptors expressed on myeloid cells 2 (TREM-2), a pro-inflammatory marker (CD86) and inducible nitric oxide synthase (iNOS) to determine the phenotypes of the microglia. We also validated microglial activation in response to BCM using a multicytokine assay (Figure 1b) and a migration assay in a microfluidic chip (Figure 1c). We found that multiple cytokines were present in BCM, which can be crucial mediators of microglia activation at the M1 state. This induced CD86 level and further led to the release of more pro-inflammatory cytokines from the stimulated microglia. Our study demonstrated the pro-inflammatory activity of BCM characterized by strong level of the CD86 marker, which illustrated that *P. gingivalis* can stimulate the M1 activation state of microglia (Figure 1d).

To test the indirect pathways, bacterial and stimulated-microglial conditioned media (BCM_L/H_ and MBCM_L/H_) were employed to investigate if *P. gingivalis* could cause neurodegeneration directly from BCM or indirectly with microglia as an intermediate. Human neural progenitor cells (ReN) were differentiated for 21 days in neural media (NM) and treated with four different conditions, including BCM_L/H_ and MBCM_L/H_. We labeled each media mixed with neural media and the BCM_L/H_ in the ratio of 100:1 as [100 NM:1 BCM_L/H_] and MBCM_L/H_ 10:1 as [10 NM:1 MBCM_L/H_] (Figure 1a). Stimulated microglial-conditioned media induced astrogliosis and neurodegeneration, as indicated by the induced level of the reactive astrocyte marker (GFAP) and neurodegenerative marker (pTau) (Figure 1e). Our results suggest that microglia are an important intermediate in neurodegeneration triggered by *P. gingivalis*.

### 2.2. Bacterial Conditioned Media Induced Microglial Inflammatory Responses

**Figure 2:**
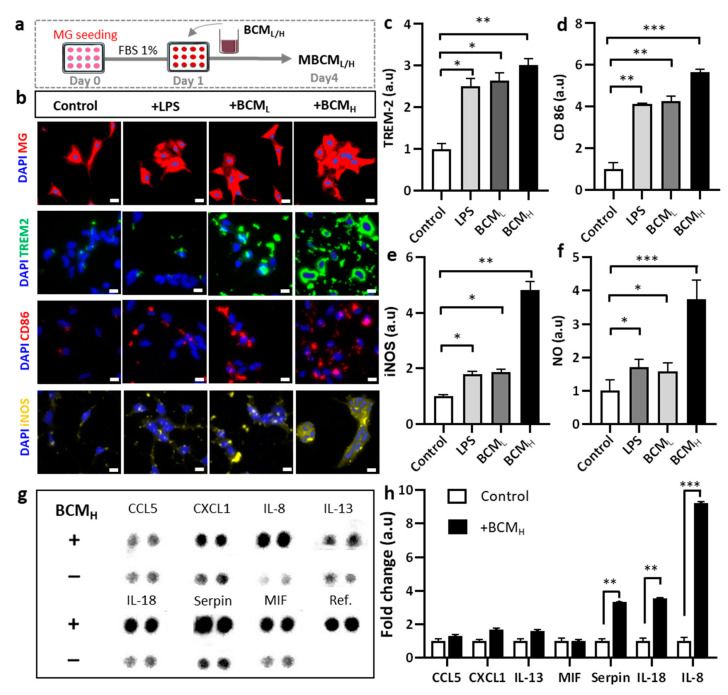
Assessment of neuro-inflammation trigged by bacterial conditioned media at two different concentrations. (**a**) Schematic diagram showing experimental timeline for neuro-inflammation assessment. (**b**) Morphological changes and immunostaining images against CD86, TREM-2 and iNOS markers of microglia cells treated with 10 ng/mL LPS, [1 BCM_L_:10 MM] and [1 BCM_H_:10 MM] in a single-culture system at day 4, scale bar 50 um. (**c**–**e**) Quantification of TREM2, CD86 and iNOS fluorescent intensity expressed by stained microglia cells. Significant differences among BCM_L_, BCM_H_ and LPS treatment, compared with the control. (**f**) Analysis of the amount of nitric oxide released by microglia cells. (**g**,**h**) Quantification of chemokines and cytokines released in microglia-stimulated conditioned media indicated increased inflammatory response from microglia cells, compared with the control. All experiments were repeated three times independently and statistically analyzed by One-way ANOVA followed by Tukey HSD test (* *p* < 0.05, ** *p* < 0.01 and *** *p* < 0.001). All data were presented as mean ± standard deviation (SD).

We investigated the microglial phenotype in response to BCM. Microglial responses to LPS, BCM_L_ and BCM_H_ were assessed, as shown in Figure 2a. We first investigated the initial response of microglia to BCM by monitoring morphological changes. LPS was used as a positive control because it is known to strongly stimulate microglia [15]. Our data showed that morphological changes were observed in non-stimulated migroglia and LPS-/BCML-/H-stimulated cells. We also observed significant aggregation of the cells treated with LPS and BCM_L/H_, compared with the non-treated cells (Figure 2b). To further validate microglial activation in response to BCM, microglia were treated with BCM and immunostained with phenotypic markers of disease associated microglia (TREM-2) and M1 type microglia (CD86) (Figure 2b). The bacterial and LPS-stimulated microglia expressed increased TREM-2 levels, compared with non-stimulated cells. In particular, the TREM-2 levels in LPS-, BCM_L_- and BCMH-stimulated cells were almost 2.5-fold, 2.7-fold and 3-fold greater than that in the control, respectively (Figure 2c). We also found that the level of CD-86 in BCM_H_-, BCM_L_- and LPS-treated microglia was significantly greater than that in the control cells (5-fold, 4-fold and 4-fold, respectively) (Figure 2d). Overall, these data indicate differences in microglial phenotype and responses between the control group and LPS/BCM_L/H_-stimulated microglia. 

To further confirm the phenotype of microglia involved in neurodegeneration, microglia cells were immunostained with inducible nitric oxide synthase (iNOS) and the level of nitric oxide (NO), which is defined as one of the causes of neuroinflammation was measured [16]. We also quantified the levels of iNOS and NO and performed statistical analysis to determine statistical significance. According to the results, BCM_H_-treated microglia expressed a significantly higher level of iNOS, which was 4.8-fold greater than the control and 2.5-fold greater than LPS-and BCL-treated microglia (Figure 2e). BCM_H_-stimulated microglia highly expressed NO, which was 3.7-fold, 2.2-fold and 2.4-fold higher than those in non-stimulated, LPS- and BCML-treated cells, respectively (Figure 2f). Next, we assessed the status of microglial inflammation by measuring multiple inflammatory cytokines in the MBCM (Figure 2g). The levels of cytokines, including IL-8, IL-18 and serpin, in BCM_H_-stimulated microglia were significantly higher, at almost 7.5-fold, 3-fold and 2.8-fold, respectively, than those in non-stimulated cells (Figure 2h). Our data suggested that BCM induced microglial pro-inflammation, which may play a detrimental role in neuroinflammation, supporting our hypothesis that BCM can trigger microgliosis during *P. gingivalis* infection.

### 2.3. Neurodegeneration Induced by Both Microglial and Bacterial Condition Media

**Figure 3:**
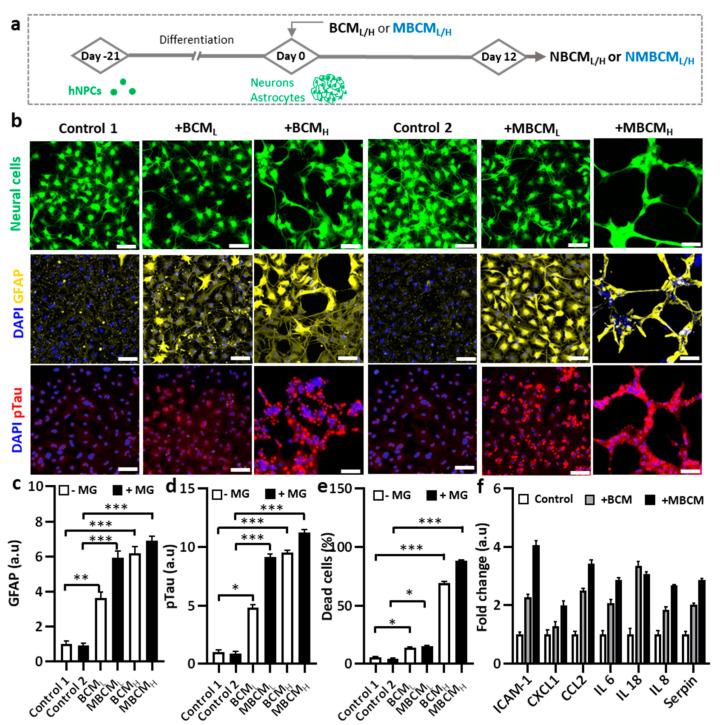
Neurodegeneration initiated by different bacteria-conditioned media (BCM_L/H_) and microglia-derived media containing bacterial metabolites (MBCM_L/H_). (**a**) Schematic diagram showing experimental timeline for astrogliosis and neurodegeneration. (**b**) Morphological changes in neurons/astrocytes (green) treated with bacterial conditioned media and microglia-stimulated conditioned media. Fluorescent images of GFAP and pTau staining of neurons/astrocytes after 12-day exposure. Scale bar, 100 μm. (**c**,**d**) Quantification of GFAP and pTau level in neurons/astrocytes. (**e**) Neural cells loss is quantified by measuring dead cell ratio. (**f**) Quantification of cytokines from astrocyte conditioned media. All experiments were repeated three times independently and statistically analyzed by One-way ANOVA followed by Tukey HSD test (* *p* < 0.05, ** *p* < 0.01 and *** *p* < 0.001). All data were presented as mean ± standard deviation (SD).

To demonstrate microgliosis-mediated neurodegeneration, co-cultured neurons and astrocyte-derived hNPCs were exposed to either BCM_L/H_ or MBCM_L/H_ (Figure 3a). We found notable morphological changes in the co-cultured neurons and astrocytes stimulated by BCM_L/H_ or MBCM_L/H_, compared with the non-treated cells (Figure 3b). In addition, GFAP levels increased in astrocytes stimulated by both BCM_L/H_ and MBCM_L/H_, representing the BCM-and MBCM-induced reactive astrocytes. We found that BCM_H_-stimulated microglial-derived media showed nearly 9-fold higher GFAP level, compared with the controls, while the BCM_H_-directed treatment caused a 7-fold increase in GFAP level (Figure 3c). In addition, treatment with MBCM_L_ and MBCM_H_ slightly increased GFAP level.

We next investigated the effect of conditioned media on neuronal cells. We found that neurons treated with MBCM_L/H_ exhibited higher pTau level than those treated with BCM_L/H_ treatment, indicating microgliosis and a higher AD risk [17]. Compared with non-stimulated neural cells, treatment with MBCM_H_ and BCM_H_ increased pTau levels by approximately 11.25 folds and 9.5 folds, respectively (Figure 3d). Our results demonstrated the association of MBCM_L_ and BCM_L_ with increase in AD risk, characterized by high pTau level. To determine cell viability, propidium iodide (PI) was used to stain dead cells, while DAPI was used to stain the nuclei of both dead and live cells. We then determined the population of dead cells (Figure 3e). The population of neurons and astrocytes treated with BCM and MBCM significantly decreased and the treatment with microglial conditioned media led to approximately 90% neuronal cell loss and the BCM_H_ treatment resulted in almost 70% neuronal death. Moreover, the low concentration of bacterial and stimulated microglia-conditioned media (BCM_L_ and MBCM_L_) reduced the population by 15%. Both low and high concentrations of BCM and MBCM increased cell death of astrocytes and neurons, while BCM_H_ and MBCM_H_ were much more detrimental to the neurons/astrocyte population at high concentrations than at low concentrations. Taken together, our results indicate that neurodegenerative microgliosis is caused by bacterial infection in AD.

Next, we performed multiple cytokine detection to identify the cytokines in stimulated neurons/astrocyte-conditioned media. Conditioned media from non-stimulated, BCM_H_-treated and MBCM_H_-stimulated neurons/astrocytes were utilized for the detection. Multiple cytokines were detected, including CXCL1, IL-8, IL-6, Serpin, IL-18, CCL2 and ICAM-1 and the level of cytokines was normalized to that in non-stimulated cells. The BCM_H_-treated cells generated more cytokines than the MBCM_H_ cells, excluding IL-18 (Figure 3f). CCL2 was ranked second in the level, at more than 3.5-fold for BCM_H_ and 2.5-fold for MBCM_H_, compared with non-stimulated cells, which clarified why NBCM_H_ recruited more microglia into the central chamber than NMBC_H_.

### 2.4. Investigating the Interaction of Astrogliosis and Microgliosis

After inducing neurodegeneration with BCM_L/H_ and MBCM_L/H_, we hypothesized that neuron-glia might have bidirectional influences on each other. Microgliosis involves the induction of loss of neurons/astrocytes (Figure 3) and astrogliosis may cause the activation of microglia, which means that microgliosis and astrogliosis may occur at the same time during infection and induce each other. 

**Figure 4:**
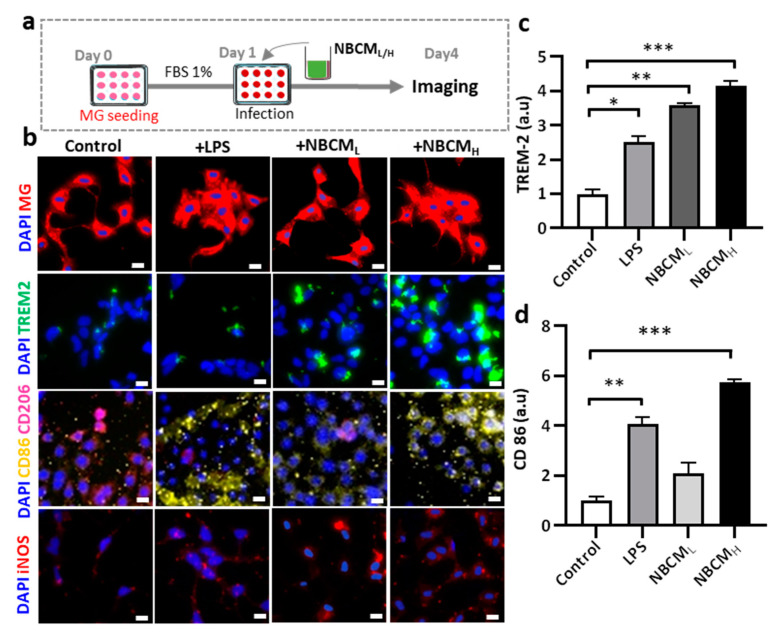

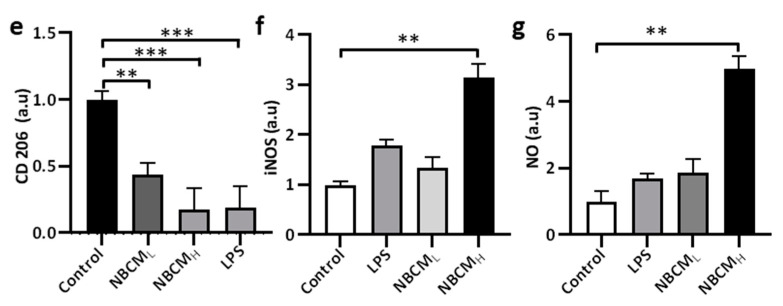
Neuron-glia interaction assessed by BCM_L/H_- and MBCM_L/H_-stimulated neurons/astrocytes conditioned media. (**a**) Schematic diagram showing experimental timeline for investigating astrogliosis and microgliosis. (**b**) Morphological changes, immunostaining of SV40 microglia cell treated with 10 ng/mL LPS, [1 NBCM:10 MM] and [1 NMBCM:10 MM] against TREM-2, CD86, CD206 and iNOS in a single culture system. (**c**–**f**) Quantification of TREM-2, CD86, CD206 and iNOS in stimulated microglia. (**g**) Analysis of nitric oxide released by stimulated microglia. All experiments were repeated three times independently and statistically analyzed by One-way ANOVA followed by Tukey HSD test (* *p* < 0.05, ** *p* < 0.01 and *** *p* < 0.001). All data were presented as mean ± standard deviation (SD).

To test our hypothesis, conditioned media of neurons/astrocytes treated with BCM_H_ or MBCM_H_ were harvested (NBCM_L/H_ or NMBCM_L/H_) and treated with the single-culture microglia (Figure 4a). To investigate the activation of microglia by astrogliosis, we monitored the morphological changes in microglia and the level of critical proteins representing microglial activation. Our results showed changes in the morphology of microglia treated with LPS and NBCM_L/H_, compared with the control. Next, microglia cells were stained against TREM-2, CD86, CD206 and iNOS to determine if stimulated microglia would exhibit a pro- or anti-inflammatory phenotype (Figure 4b). We found that microglia treated with NBCM showed higher TREM-2 level than those treated with LPS (Figure 4c). Interestingly, microglia treated with neurons/astrocyte-conditioned media co-expressed CD86 and CD206. NBCM_H_-treated microglia cells showed a higher level of CD86 protein than LPS-treated and non-stimulated microglial cells, but at low concentration, a low CD86 level, which was not statistically different from that of non-stimulated cells, was observed (Figure 4d). In particular, the NBCM_H_-stimulated microglia showed a 1.4-fold increase in CD86 level, in comparison with LPS-treated microglia, but a 5.7-fold increase, in comparison with non-stimulated microglia. With regard to the effect of low and high concentrations, NBCM_H_ caused a 2.8-fold increase in CD86 level, compared with NBCM_L_. Consistently, non-stimulated microglia showed higher level of anti-inflammatory protein markers than the treated cells, while microglia stimulated with LPS showed the lowest level of CD206 (Figure 4e). The level of CD206 in NBCM_L_-treated microglia cells was 1.4-fold lower than that in non-stimulated cells. LPS and NBCM_H_ caused a decrease in CD206 level, which was almost 5-fold lower than that in the control. Briefly, NBCM_H_ induced pro-inflammatory protein markers better than NBCM_L_, but greater anti-inflammatory activity was induced at the low concentration of conditioned media. In addition, microglia treated with neurons/astrocyte conditioned media significantly activated the iNOS pathway, compared with non-stimulated cells; in particular, NBCM_H_ showed the best efficacy in the activation of iNOS, which was 3.2-fold higher than that of the control (Figure 4f). Consistently, NBCM_H_-treated microglia generated higher levels of NO than NBCM_H_-treated (2-fold) and LPS-treated cells (2.4-fold) (Figure 4g). Overall, our data showed that BCM-stimulated astrogliosis could also induce microglial activation.

### 2.5. Microglial Migration and Investigation of New Bacterial Metabolites

**Figure 5:**
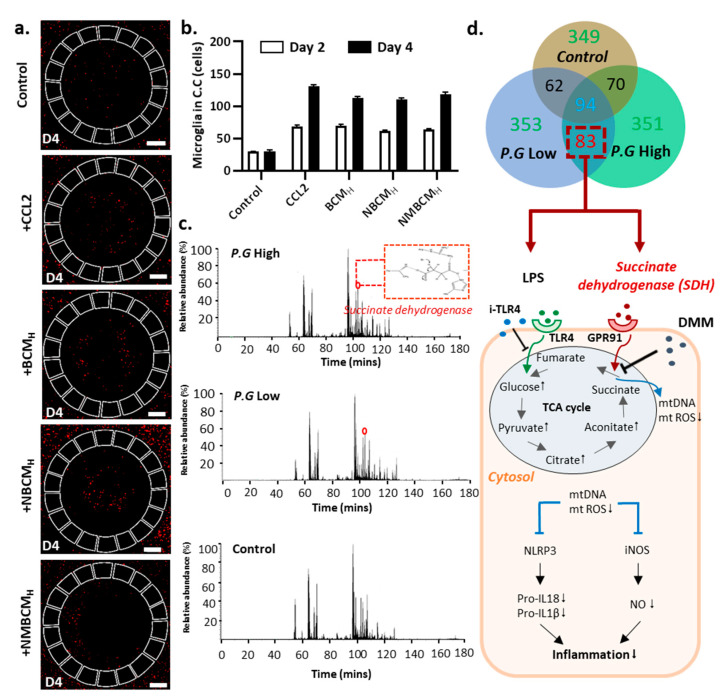
Microglial recruitment in microfluidic chip and investigation of new bacterial metabolites from the bacteria-conditioned medium. (**a**) Fluorescent images of microglial migration in brain-on-chip upon different mediators. (**b**) Quantification of recruited microglial cells into the central chamber at day 2 and day 4. (**c**) The results of liquid chromatography–mass spectrometry (LC-MS) analysis of bacterial conditioned medium (high and low concentrations) and control. (**d**) Total compounds found in three samples, high/low concentration BCM and medium. The proposed cellular mechanism of how the inhibition of succinate dehydrogenase could reduce neuroinflammation in microglia. All experiments were repeated three times independently and statistically analyzed by One-way ANOVA followed by Tukey HSD test. All data were presented as mean ± standard deviation (SD).

We utilized our established microfluidic model to assess microglial recruitment induced by three different conditioned media from previous experiments, including bacteria-conditioned media, stimulated microglia and stimulated neurons/astrocyte-derived culture media (Figure 1b). Microglia were seeded in the angular chambers and recruitment mediators were seeded in the central chamber. The recruited microglial cells were imaged and processed, which showed an increase in the number of cells on day 4 (Figure 5a). On day 2, the number of recruited microglia by CCL2 and the three conditioned media was similar. However, slight differences occurred at day 4 with CCL2 being the most attractive (130 cells), followed by NBCM_H_ (118 cells), while BCM_H_ and NMBCM_H_ achieved the same results (approximately 110 cells). CCL2 is one of the multiple cytokines detected in BCM_H_-and MBCMH-stimulated neurons/astrocytes and, hence, it is reasonable to explore its recruitment ability.

We next investigated how the recruited microglia contributed to neuroinflammation. To understand the underlying mechanisms of neuroinflammation driven by bacterial metabolites, we performed LC-MS analysis to investigate potential compounds that induce inflammation in the bacterial conditioned medium. Three different media, high and low concentrations of BCM and control, were analyzed using LC-MS (Figure 5c). After analysis, 349, 353 and 351 compounds were detected in the control, BCM_H_ and BCM_L_, respectively. Succinate dehydrogenase (SDH) is one of the 83 compounds found in both BCM_H_ and BCM_L_ and it is known to increase succinate oxidation, which upregulates the levels of nitric oxide and pro-inflammatory cytokines (Figure 5d). Upon LPS stimulation, microglia may induce glycolysis and increase the oxidation of succinate by the activities of SDH [18]. Co-stimulation might be the critical factor associated with the enhancement of microgliosis, which results in the loss and dysfunction of neuronal cells due to the upregulation of nitric oxide and pro-inflammatory cytokines. The inhibition of SDH with dimethyl malonate (DMM) may reduce the levels of mtDNA and mtROS released from damaged mitochondria, resulting in a decrease in inflammatory responses through the downregulation of pro-inflammatory cytokines and NO.

### 2.6. Inhibition of SDH and LPS Reduces Microglial Inflammatory Responses

**Figure 6:**
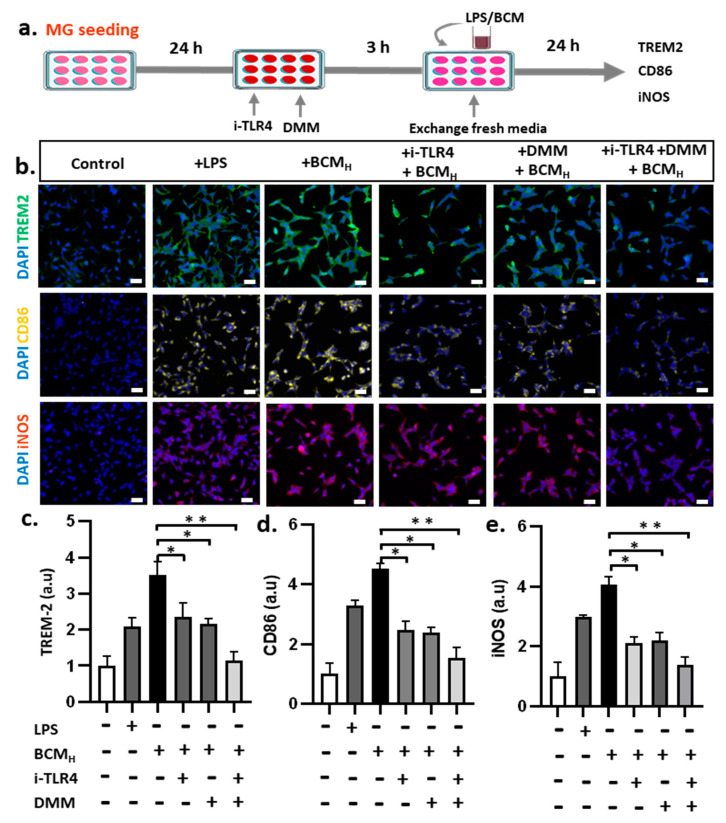
Inhibition of succinate dehydrogenase and LPS reducing microglial inflammatory responses. (**a**) Schematic showing experimental timeline for inhibition assay. (**b**) Immunostaining images against TREM-2, CD86 and iNOS markers of microglia cells. (**c**–**e**) Quantification of TREM2, CD86 and iNOS fluorescent intensity expressed by stained microglia cells. All experiments were repeated three times independently and statistically analyzed by One-way ANOVA followed by Tukey HSD test (* *p* < 0.05, ** *p* < 0.01). All data were presented as mean ± standard deviation (SD).

We explored the cell-permeable molecule, DMM, which is rapidly hydrolyzed within the cell to generate malonate, a potent competitive inhibitor of SDH-induced succinate oxidation [19]. To test our hypothesis, we inhibited human TLR4 (hTLR4) to reduce LPS uptake and utilized DMM as an inhibitor of SDH upon stimulation of microglial cells by LPS and BCM (Figure 6a).

To assess microglial responses to stimulators with single/co-inhibitors, the cells were immunostained with several protein markers, including TREM-2, CD-86 and iNOS (Figure 6b). TREM-2 level was decreased in cells treated with BCM_H_ + hTLR4/DMM (Figure 6c). In comparison with the control, LPS and BCM_H_ led to a 2-fold and 3.5-fold increase in TREM-2 level, respectively. After single and co-inhibition with hTLR4 and DMM, the level of TREM-2 decreased in treatments stimulated by BCM_H_. In particular, single inhibition with hTLR4 or DMM led to an almost 1.75-fold decrease in TREM-2 level from 3.5-fold to nearly 2-fold greater than that in the control. When co-inhibited with TLR4 and DMM, TREM-2 level was nearly the same as that in non-stimulated microglial cells. Similarly, there were significant changes in the level of CD86 in the inhibition treatments. Both individual and combined inhibition resulted in a decrease in CD86 levels upon BCM_H_ stimulation (Figure 6d). Microglial cells treated with LPS and BCM_H_ showed nearly 3-fold and 4.5-fold greater level of CD86, compared with non-stimulated cells, with significant differences. Upon inhibition by TLR4 and DMM, BCM_H_-stimulated microglia demonstrated an almost 2-fold decrease in CD86 level in treatment with only TLR4 or DMM and a 2.5-fold decrease in combined inhibition. In addition, there were no significant differences in the cells treated with inhibitors and the control. Consistently, LPS-and BCMH-stimulated microglial cells increased the level of iNOS by almost 2.75 folds and 4 folds, respectively, compared with the control (Figure 6e). When we inhibited with only TLR4 or DMM upon BCM_H_ stimulation, iNOS level was reduced from 4 fold to almost 2.5 fold in the individual inhibition and 1.5 fold in combined TLR4/DMM inhibition, compared with the control, without statistical significance. Based on this evidence, we can conclude that LPS and SDH are co-stimulators of neuroinflammation in microglia with BCM_H._ The inhibition of either LPS or SDH slightly reduced microglial inflammatory responses, whereas combined inhibition showed a more significant reduction in microglial activation.

## 3. Discussion

Our study not only illustrates *P. gingivalis*-induced neurodegeneration as previously reported [13], but also demonstrates the critical role of microglia in response to bacterial infection in the central nervous system. This study directly connects three factors involved in AD, including bacterial infection, neuroinflammation and neurodegeneration, using human neural cell culture platforms. Microglia are resident innate immune cells in the brain and are responsible for infection control and clearance of external pathogens [20]. *P. gingivalis* infection has been linked to microglia and neurodegeneration in AD in a mouse model [21]; however, the mechanism through which *P. gingivalis* alters microglial activity and causes neuroinflammation and neurodegeneration in humans remains unknown.

Neuroinflammation induced by bacterial or viral infection is considered a crucial player in the pathogenesis of AD [22]. TREM-2 is a protein expressed at the early and middle stages of AD and acts as an Aβ receptor. TREM-2 level is upregulated in plaque-associated microglia in both mice and humans [23]. We demonstrated that BCM triggered the level of TREM-2 in human microglia, which proved its association with AD. Microglia in the brain are highly plastic and can adopt distinctive phenotypes, including the classically activated (M1) and alternatively activated (M2) states in response to various simulations [24]. The M1 state of microglia is characterized by the production of pro-inflammatory cytokines and induced level of surface protein markers as well as inducible nitric oxide synthase, leading to NO generation [25]. The results of our study showed that *P. gingivalis* induced high level of CD86 protein (M1 marker) and iNOS as well as NO production, while multiple pro-inflammatory cytokines, including IL-8, IL-18, MIF and Serpin, which facilitated the inflammatory process, were also expressed. Interleukin-8 (IL-8) is a pro-inflammatory cytokine produced by microglia, neurons and astrocytes and it is highly expressed in the serum, CSF and brain of AD patients [26]. A previous study reported an increase in IL-8 production in cultured microglia obtained post-mortem from AD and non-demented individuals [27]. In addition, CSF levels of IL-8 were significantly elevated in patients with mild cognitive impairment and in individuals diagnosed with AD, compared with age-matched controls [28]. 

Astrocytes can also initiate neuroinflammation, leading to neurodegeneration in Alzheimer’s disease [29]. Astrocytes, the most abundant cells in the central nervous system, can be reactive during acute infection for clearance of pathogens and can induce neurological impairment [30]. Stimulation of reactive astrocytes can occur directly with *P. gingivalis* or through indirect pathways with microglia as an intermediate, which was demonstrated with bacterial conditioned media and bacterial-stimulated microglia condition media in our work. We observed the level of glial fibrillary acidic protein (GFAP), a common feature of reactive astrocytes, induced by bacterial and bacterial-stimulated microglia-conditioned media. Multiple cytokines detected in bacterial conditioned media are potential inflammatory mediators for astrocyte activation. A previous study indicated that iPSC-derived astrocytes stimulated by IL-1β generated much more IL-8 and IL-6, compared with non-stimulated astrocytes [31], which is consistent with our results in which IL-8 and IL-6 were detected in stimulated neurons/astrocyte-conditioned media. In addition, microglia treated with bacterial conditioned media induce the level of several pro-inflammatory cytokines, such as IL-8, IL-18 and MIF, further leading to the formation of reactive astrocytes. A similar study demonstrated that pro-inflammatory cytokines produced by LPS-stimulated microglia are differentiation factors that promote the A1 phenotype of astrocytes, which are abundant in various human neurodegenerative diseases [32]. 

In this study, neuroinflammation-derived factors induced neurodegeneration after long-term exposure. Neurodegenerative microgliosis is associated with the loss of neuronal cells, alterations of phenotype and the level of pTau protein, which is a critical hallmark of AD. The generation of IL-8 from stimulated microglia and astrocytes can be the main factor in neurodegeneration due to its neurotoxic effects. Cell death was induced in neuronal cells treated with IL-8, which was associated with changes in pro-apoptotic proteins and inflammatory mediators [33]. In particular, bacterial-stimulated microglia demonstrated more significant results in neuronal loss than direct bacterial conditioned media treatment, which could be due to the high level of IL-8 and other inflammatory cytokines from stimulated microglia and astrocytes. This study strongly demonstrated the critical role of microglia in neuroinflammation and neurodegeneration during *P. gingivalis* infection and pointed out that IL-8 released from stimulated microglia and astrocytes is the major neurotoxic cytokine in *P. gingivalis* infection, which contributes to the understanding of the role of the mouth-brain axis in AD.

## 4. Materials and Methods

### 4.1. Preparation of Bacterial Conditioned Medium

*P. gingivalis* was proliferated in Brain Heart Infusion (BHI) broth supplemented with hemin (0.5 mg/mL) and vitamin K (0.5 mg/mL) until reaching 2.55 × 10^9^ CFU/mL (BCM_L_) and 7.62 × 10^10^ CFU/mL (BCM_H_). Bacterial cells were removed from the final conditioned media by centrifuging and filtering through 0.2 µm membranes. The samples were stored at −80 °C for further experiments.

### 4.2. Liquid Chromatography-Mass Spectrometry (LC-MS) Analysis

The prepared samples were resuspended in 0.1% formic acid in water and analyzed using a Q-Exactive Orbitrap hybrid mass spectrometer (Thermo Fisher Scientific, Waltham, MA, USA) and an Ultimate 3000 system (Thermo Fisher Scientific, Waltham, MA, USA). We used a 2 cm × 75 μm ID trap column packed with 3 μm C18 resin and a 50 cm × 75 μm ID analytical column packed with 2 μm C18 resin peptides depending on the hydrophobicity of the peptides. A data-dependent acquisition method was adopted and the top 10 precursor peaks were selected and isolated for fragmentation. Ions were scanned at high resolution (70,000 in MS1, 17,500 in MS2 at *m*/*z* 400) and the MS scan range was 400–2000 *m*/*z* at both MS1 and MS2 levels. Precursor ions were fragmented using 27% normalized collisional energy (NCE). Dynamic exclusion was set to 30 s. For proteome data analysis, MS/MS data obtained from the Thermo Q-Exactive instrument were converted to mzXML using MSConvert for searching in Andromeda of MaxQuant (version 1.5.8.3) (Martinsried, Germany). A cut-off probability score of less than 1% was used for the FDR. Information about the identified peptides and proteins was aligned using the mass of the charge state, retention time and peak area.

### 4.3. Preparation of Membrane Stained-Microglia

SV40 immortalized human adult microglia cells were purchased from Applied Biological Material Inc. (Montreal, Canada) and grown in T25 flasks (SPL Life Sciences Co., Ltd., Pocheon-si, Korea) containing microglia medium (MM), which comprised Pigrow III Medium (ABM Inc., Richmond, BC, Canada) supplemented with 10% FBS (Life Technologies, Grand Island, NY, USA) at 37 °C in the presence of 5% CO_2_. The culture medium was changed every 2 days until the cells reached confluence. The membrane of the microglia was stained with red dye (PKH26PCL Red Fluorescence Cell Linker, Sigma Aldrich, St. Louis, MO, USA). The cells were detached using trypsin EDTA (Thermofisher Scientific, Waltham, MA, USA) and centrifuged at 1300 rpm for 3 min to collect the cells. The pellets were instantly re-suspended in a mixture containing 1 mL of Diluent B (Sigma Aldrich, St Louis, MO, USA) and 4 μL of dye solution. The mixture of cell/dye was incubated at room temperature for 10 min in the dark and periodically mixed to obtain uniform staining, after which 1 mL of 1% BSA in PBS was added to stop the dying process. Undyed cells and excess dye solution were eliminated by centrifuging twice at 1300 rpm for 3 min.

### 4.4. Microglial Cells Culture Platform

Membrane-stained microglia cells were seeded into Matrigel (BD Biosciences, San Jose, CA, USA)-coated 96 well-plates (Thermo Scientific, Seoul, Korea) at a density of 5000 cells/well. Positive controls were treated with 10 ng/mL LPS, while the treatment groups were treated with BCMs at 24 h after seeding. BCM was mixed with MM at a 1:10 ratio and microglial cultures were collected every 2 days, until the wells were confluent, for further analysis. Two groups of MBCM were collected for subsequent experiment: MBCM_L_ and MBCM_H_.

### 4.5. Proliferation of Neurons/Astrocytes

Human neural progenitor cells (ReN) were purchased from EMD Millipore (Billerica, MA, USA) and grown in 1:100 Matrigel-coated T25 flasks (SPL Life Sciences Co., Pocheon-si, Korea). The flasks contained DMEM/F12 medium (Life Technologies, Grand Island, NY, USA) supplemented with 2 mg heparin (Stemcell Technologies, Vancouver, BC, Canada), 2% (*v*/*v*) B27 (Life Technologies, Grand Island, NY, USA), 20 mg EGF (Sigma-Aldrich, St Louis, MO, USA), 20 mg bFGF (Stemgent, Cambridge, MA, USA) and 1% (*v*/*v*) PSA antibiotic solution (Lonza, Hopkinton, MA, USA). The culture medium was changed every 2 days until the cells reached confluence. 

### 4.6. Differentiation of Neurons/Astrocytes

ReN cells were loaded into 96 well-plates coated with matrigel (1:100) at a density of 10^4^ cells/well supplemented with differentiation media for neuronal cells (NM). The differentiation media consisted of DMEM/F12 media (Life Technologies, Grand Island, NY, USA) supplemented with 2 mg heparin (Stemcell Technologies, Vancouver, BC, Canada), 2% (*v*/*v*) B27 (Life Technologies, Grand Island, NY, USA) and 1% (*v*/*v*) antibiotic solution (Lonza, Hopkinton, MA, USA). After three weeks of differentiation, the cells were treated with bacteria and microglia-stimulated conditioned media. The experiment involved four treatment groups and two control groups.

### 4.7. Microfluidic Chip Fabrication

The microfluidic chip comprised three compartments, including an acrylic sheet at the top, a PDMS layer and a glass side at the bottom, which were fabricated by photolithography and soft lithography, as described in our previous study [34]. In brief, a mold was created by patterning negative photoresist SU-8 50 and SU-8 100 (MicroChem, Newton, MA, USA) onto a 4 inch silicon wafer to create microchannels with heights of 50 μm and 100 μm for cell migration and chemoattraction, respectively. The platform was generated by pouring a mixture of polydimethylsiloxane (PDMS) base and curing reagent at a 10:1 ratio (Sylgard 184 A/B, Dow Corning, Midland, MI, USA) into the mold, followed by vacuuming for 20 min to remove bubbles and incubation at 60 °C for at least 4 h for full curing. Next, 2 mm holes were made into PDMS sheets. Acrylic thickness of 6 mm was prepared using a laser cutter (Zing 24, Epilog Laser, Golden, CO, USA) to array holes for the media reservoir. To combine the PDMS layer and acrylic, uncured PBMS:curing agent (10:1) was utilized as glue, followed by incubation at 60 °C overnight. The glass slides and the assembled PDMS sheets were exposed to oxygen plasma at 50 mW and 5 cm for 30 s (PX-250, March Plasma System, Petersburg, FL, USA) and bonded together. 

### 4.8. Microglia Loading into the Microfluidic Chip

SV40 cells were membrane-stained, as previously described, at a cell density of 5 × 10^5^ cells/mL. Cells (10 µL) were injected into each platform from the angular chambers, followed by incubation for 1 h at 37 °C for cell adherence. Next, 100 μL of culture medium supplemented with 1% FBS was added into two angular reservoir chambers. Microglial culture media supplemented with 1% FBS (MM 1%) and CCL2 (10 ng/mL) in 1% MM were used as negative and positive controls, respectively. The dilution ratios of BCM_H_ (100 fold) and NBCM_H_ and NMBCM_H_ (10 fold) synchronized with the ratio of bacterial conditioned media in the central chamber.

### 4.9. Fluorescent Immunostaining

When the cells reached confluence, they were washed twice with 1× PBS and fixed for 15 min at room temperature with 4% paraformaldehyde solution (Biosesang, Seongnam-si, Korea) and then rinsed twice with phosphate-buffered saline with 0.1% Tween^®^20 (PBST) twice to remove the excess fixing solution. For permeabilization, the cells were incubated with 0.1% Triton-X 100 in PBST for 15 min at RT and the blocking step was followed by incubation in 3% BSA (BSA 100, Bovogen, Melbourne, VIC, Australia) for 1 h at room temperature. The samples were incubated with CD-86 (Ab239075, Abcam, Cambridge, MA, USA) and CD 206 (Abx140462, Abbexa Ltd., Cambridge, UK), iNOS (PA1-036, ThermoFisher scientific, Seoul, Korea) and TREM-2 (AF1828, R&D System, Minneapolis, MN, USA) for microglia and with pTau (MN1020, ThermoFisher Scientific, Seoul, Korea) and GFAP (AB5541, EDM Millipore, Billerica, MA, USA) for neurons/astrocytes overnight at 4 °C. Next, the samples were washed three times before incubation with secondary antibodies and DAPI for 1h at RT. The samples were washed twice with PBST and the images were captured.

### 4.10. Viability Assay

Cells were stained with propidium iodide (P1304MP, Thermo Fisher Scientific, Seoul, Korea) for 15 min to detect dead cells. Next, the cells were washed thrice with PBS 1× to remove the excess dye. The cells were fixed in 4% paraformaldehyde solution, followed by DAPI staining for total nucleus recognition. PI and DAPI-stained cells were detected using a fluorescent microscope and the percentage of dead cells per total was determined using ImageJ software (Wayne Rasband, NIH).

### 4.11. Nitric Oxide Measurement

Nitric oxide (NO) released from microglia was measured using DAF-FM™ diacetate as an NO indicator (D-23844, Thermo Fisher Scientific, Seoul, Korea). A 5 nM stock solution was prepared by dissolving 50 μg of DAF-FM™ diacetate in 20 μL of high-quality anhydrous DMSO and the stock was diluted to 10 μM in cell culture media. Microglia were prepared in a 96 well-plate and washed with PBS 1× once before exposure to the NO indicator. Next, the cells were incubated with 10 µM DAF-FM™ diacetate at 37 °C for 60 min, followed by washing twice with PBS 1× to remove the excess probe. The old medium was replaced with fresh medium, followed by incubation for an additional 30 min to allow complete de-esterification of the intracellular diacetates. A fluorescent microscope was used to detect FITC signals from NO-indicated microglia.

### 4.12. Detection of Multiple Cytokines

A human multiple cytokine array kit was purchased from R&D Systems, Minneapolis, MN, USA (Catalog#ARY005) and utilized according to the manufacturer’s protocol provided by the company. Briefly, the captured antibodies were spotted in duplicate on nitrocellulose membranes. Each sample (0.5 mL) was mixed with a cocktail of biotinylated detection antibodies. The sample/antibody mixture was then incubated with the array overnight at 4 °C. Any cytokine/detection antibody complex was bound by its cognate-immobilized capture antibody on the membrane. Streptavidin-horseradish peroxidase (HRP) and chemiluminescent detection reagents were added. A signal is produced proportional to the amount of cytokine bound. Chemiluminescence was detected in the same manner as the western blot and the results were quantified using ImageJ software (Wayne Rasband, NIH).

### 4.13. Inhibition Assay

SV40 immortalized human adult microglia cells were seeded into matrigel-coated 96 well-plates at a density of 5000 cells/well. HumanTLR4 neutralization (Cat#mabg-htlr4) was purchased from InvivoGen (San Diego, CA, USA) and dimethyl malonate (Cat# 136441) was purchased from Sigma-Aldrich (St Louis, MO, USA). SV40 cells were pretreated with human TLR4 neutralization (hTLR4, 10 μg/mL) or dymethyl malonate (DMM 5 mM) for 3 h before being stimulated with LPS (10 ng/mL) and [10 MM:1 BCM_H_] for 24 h.

### 4.14. Fluorescence Imaging

All fluorescent images were taken using a Nikon TiE microscope (Nikon, Japan) with a heated incubator at 37 °C and 5% CO_2_. Large images were taken with a 10× objective and 7 × 7 fields.

### 4.15. Statistical Analysis

All the data were analyzed using GraphPad Prism software (version 6) (San Diego, CA, USA). The differences among treatments were statistically tested using one-way analysis of variance followed by a post-hoc Tukey test. All data were normalized by control and presented as mean ± standard deviation and *p* < 0.05 indicated statistical significance. Symbols (*, **, ***) denote significant differences between the groups.

## Data Availability

The authors hereby declare that the data of this study will be presented upon request from the corresponding author.
